# Interval breast cancer rates for tomosynthesis vs mammography population screening: a systematic review and meta-analysis of prospective studies

**DOI:** 10.1007/s00330-024-11085-9

**Published:** 2024-10-03

**Authors:** Sol Libesman, Tong Li, M. Luke Marinovich, Anna Lene Seidler, Alberto Stefano Tagliafico, Nehmat Houssami

**Affiliations:** 1 The NHMRC Clinical Trial Centre, The University of Sydney, A Joint Venture with Cancer Council NSW, Sydney, NSW Australia; 2 The Daffodil Centre, The University of Sydney, A Joint Venture with Cancer Council NSW, Sydney, NSW Australia; 3 Radiology Section, Department of Health Sciences (DISSAL), University of Genova, Genoa, Italy; 4 Department of Radiology, IRCCS—Ospedale Policlinico San Martino, Genoa, Italy; 5 Sydney School of Public Health, Faculty of Medicine and Health, University of Sydney, Sydney, NSW Australia

**Keywords:** Breast cancer, Cancer screening, Digital breast tomosynthesis, Interval cancer, Mammography

## Abstract

**Objectives:**

We aimed to synthesise evidence from prospective studies of digital breast tomosynthesis (DBT) screening to assess its effectiveness compared to digital mammography (DM). Specifically, we examined whether DBT reduces interval cancer rates (ICRs) in population breast cancer screening.

**Materials and methods:**

We performed a systematic review and meta-analysis of DBT screening studies (identified from January 2013 to March 2024). We included both RCTs and non-randomised prospective studies that used an independent comparison for our primary outcome ICRs. The risk of bias was assessed with QUADAS-2. We compared the ICR, cancer detection rate (CDR), and recall rate of DBT and DM screening using random effects meta-analysis models. Subgroup analyses estimated outcomes by study design. Sensitivity analyses estimated absolute effects from relative effects.

**Results:**

Ten prospective studies (three RCTs, seven non-randomised) were eligible; all had a low risk of bias. There were 205,245 DBT-screened and 306,476 DM-screened participants with follow-up for interval cancer data. The pooled absolute ICR did not significantly differ between DBT and DM: −2.92 per 10,000 screens (95% CI: −6.39 to 0.54); however subsequent subgroup analysis indicated certain study designs may have biased this ICR estimate. Pooled ICR from studies that only sampled groups from the same time and region indicated DBT led to 5.50 less IC per 10,000 screens (95% CI: −9.47 to −1.54). Estimates from subgroup analysis that compared randomised and non-randomised trials did not significantly differ.

**Conclusion:**

This meta-analysis provides suggestive evidence that DBT decreases ICR relative to DM screening; further evidence is needed to reduce uncertainty regarding ICR differences between DBT and DM.

**Key Points:**

***Question*** *Does DBT have long-term benefits over standard DM*?

***Finding*** *We find suggestive evidence in our primary analysis and stronger evidence in a follow-up analysis that DBT reduces interval cancers*.

***Clinical relevance*** *This meta-analysis provides the first indication that DBT may detect additional cancers that are clinically meaningful, based on suggestive evidence of a reduction in ICR. This finding does not preclude the simultaneous possibility of overdiagnosis*.

**Graphical Abstract:**

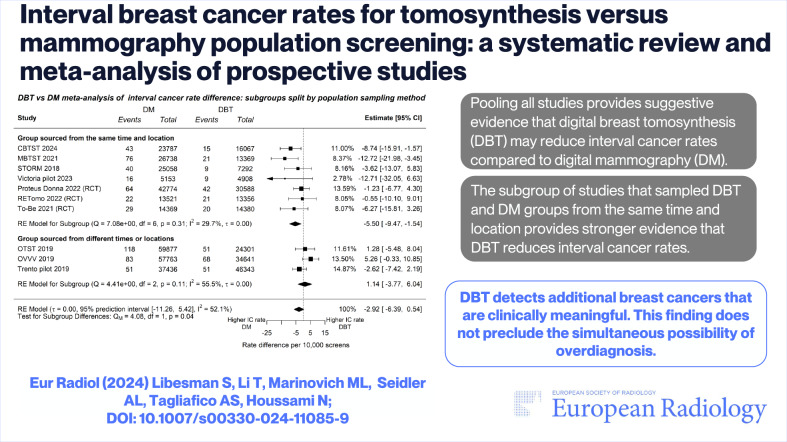

## Introduction

Digital mammography (DM) is currently the standard imaging method for breast cancer (BC) screening in most settings. Over the past decade, digital breast tomosynthesis (DBT) has been increasingly considered as a replacement for DM. By reducing overlapping breast tissue as compared to DM, DBT may allow for improved visualisation of malignancy [1]. Empirically, relative to DM, DBT has been found to increase BC detection, particularly in 2-yearly screening practice, with no consistent evidence of a substantial difference in recall rate [2–5]. However, it is currently unclear whether the increases in DBT cancer detection confer further reduction in BC mortality (relative to DM), or whether it is mostly adding overdiagnosis [6, 7]. Understanding the collective benefits and harms of DBT relative to DM is an imperative step in evaluating which screening technology should be recommended for future screening practice—as it currently stands DBT has conditional recommendation in organised breast screening programs due to uncertainty of the evidence [8].

Because BC mortality as an endpoint requires very large trials and extended follow-up, a surrogate measure of screening effectiveness such as interval cancer rate (ICR) has been proposed [9]. When assessing a new screening modality, a reduction in ICRs at follow-up (relative to standard screening) is an indication that the new method is detecting cancers that would have clinically progressed between scheduled screening examinations [10]. Recent individual participant data (IPD) meta-analysis of non-randomised studies that evaluated DBT vs DM in population screening settings, did not find evidence of a reduction in interval cancers for DBT despite an increase in cancer detection [2, 11]. Although IPD meta-analyses are considered the gold standard in evidence synthesis, an important limitation of the previous analysis was that few studies were included, and none were randomised. Random allocation to intervention groups removes selection bias and increases the likelihood of balancing any measured or unmeasured participant characteristics that may affect outcomes [12].

We conducted a systematic review with meta-analysis to assess the impact of DBT vs DM on ICR in population screening across all available prospective studies including emerging evidence from the randomised controlled trials (RCT). If non-randomised studies do not appear biased, pooling data across both design methods allows the estimation of ICR with increased precision. This is particularly important when analysing interval cancers, which are rare events. By further investigating the effectiveness of DBT, this paper is the first review of high-quality RCT and prospective study evidence to examine whether DBT decreases ICR relative to DM.

## Methods

This systematic review and meta-analysis were prospectively registered on PROSPERO (CRD42023462933). The publication followed the PRISMA guidelines for study-level meta-analyses (Supplementary Table 1).

### Eligibility criteria

Studies were included in the review and meta-analysis if they met all the pre-specified inclusion criteria. Eligible studies screened women aged ≥ 40 years; investigated DBT (interpreted alone, or in conjunction with synthetic 2D images or acquired 2D), in comparison with standard DM for screening; had a prospective design and prospective recruitment or inclusion of participants for DBT screening; reported rate or count data for BC detection and interval cancers. Designs included were randomised controlled trials, trials that contained a single arm that prospectively collected DBT compared against a retrospective cohort at follow-up for the purpose of ICR, and prospective cohort studies.

Studies were excluded from the review if they had any of the following: retrospective design, predominantly based on participants with increased risk of BC or based on symptomatic women (i.e. not population screening), or used cancer-enriched mammography datasets.

### Search strategy

We conducted a systematic search of the biomedical literature to identify studies that meet our eligibility criteria. The search period ranged from January 2013 (when DBT was first trialled in screening research) up to March 2024. The databases used for record identification were EMBASE, Pre-MEDLINE, the Database of Abstracts of Reviews of Effects (DARE), Health Technology Assessment (HTA), NHS Economic Evaluation Database (NHSEED), ACP Journal Club, and Cochrane. Keywords and headings searched included the terms “breast cancer”, “tomosynthesis”, “DBT”, “3D mammography”, and “screening”. The full strategy is available in Supplementary Table 3.

### Selection of studies for inclusion and data extraction

Once all records were identified by the search, the titles and abstracts were screened by one author (S.L.), with another author checking a subset of 10% of abstracts (T.L.). Any conflicts were resolved by consensus, otherwise a third author (N.H.) arbitrated. Full texts were double-screened, and any conflicts were resolved through meetings to achieve consensus (S.L. and T.L.). Study-specific data were extracted independently by two authors (S.L. and T.L.). Any disagreements were flagged and resolved through consensus, and if not resolved, a third author (N.H.) arbitrated.

#### Data items

Study-level information extracted from each study included: country (region); study design (randomised control or non-randomised prospective study); screening start and finish dates; screening interval (annual, biennial, or combinations); DBT intervention (number of views); method of reading (single or double-reading); method or rule for deciding recall (including scoring used if applicable); and method or rule for resolving discordant double-reading (where applicable). Participant age at screening (median and IQR) was also extracted.

Extracted endpoints were: the number of recalls (and/or recall rate) and number of screens; the number of BCs detected and number of screens (and/or cancer detection rate (CDR)); and the primary outcome ICR (and/or record of interval cancer diagnosis within the screening interval and number of screens).

### Statistical analysis

#### Effect measures and outcomes

Study and endpoint characteristics were summarized descriptively within studies and study groups (DBT and DM) using the mean (SD) for continuous variables and counts (percent) for categorical variables. The primary outcome was ICR per 10,000 screens. Secondary outcomes were quantified as CDR per 10,000 screens, and recall rate (%). All outcomes were stratified by study design (RCT vs non-randomised study).

#### Risk of bias

Quality appraisal was undertaken using the Revised Quality Assessment of Diagnostic Accuracy Studies (QUADAS-2) checklist, adapted for application to studies in the screening setting [4]. Scoring was conducted by one author (S.L.) and reviewed by a second author (N.H.). Disagreements were resolved by discussion and consensus.

#### Data synthesis

Study-specific recall rates, CDRs, ICRs and pooled estimates were displayed on forest plots. The pooled estimates were summarised with means and 95% confidence intervals (CI) [13]. We compared screening modalities (DBT and DM) by pooling rate differences (RD) and rate ratios (RR) in inverse variance-weighted random effects meta-analysis models. This approach incorporates a heterogeneity parameter when pooling estimates. Pooling risk differences may be biased by varying baseline risk across different study contexts [14], thus we conducted sensitivity analyses that estimated RD by transforming the pooled relative effect into absolute estimates using the assumed baseline risk for DM (the reference test). This was done using simulation to account for baseline uncertainty [15]. The baseline risk for DM was estimated for a given outcome by meta-analysing women screened with DM included in the present review.

If studies reported both paired and unpaired outcome data, we analysed the unpaired data because only this approach supports the analysis of ICR. We performed a subgroup analysis that compared study designs (RCT vs non-randomised). We performed a post-hoc sensitivity analysis excluding studies that may have introduced bias by sourcing DM cohorts (for IC data) from different populations (either different time periods or different regions). Differences were evaluated with *Q*_m_ tests and through visual inspection of the forest plots. Subgroup *p*-values were two-tailed with an alpha (significance cut-off) set at 0.05. Heterogeneity was examined through between-study standard deviation (τ), and 95% prediction intervals (PI), which capture the likely range of effect sizes if a single study were to be selected at random from a population of studies akin to the ones included in the analysis, and inconsistency, the proportion of heterogeneity compared to sampling error (*I*^2^). All analyses were conducted using the metafor package (version 3.0-2) [16] for R (version 4.3.3) [17].

## Results

**Fig. 1:**
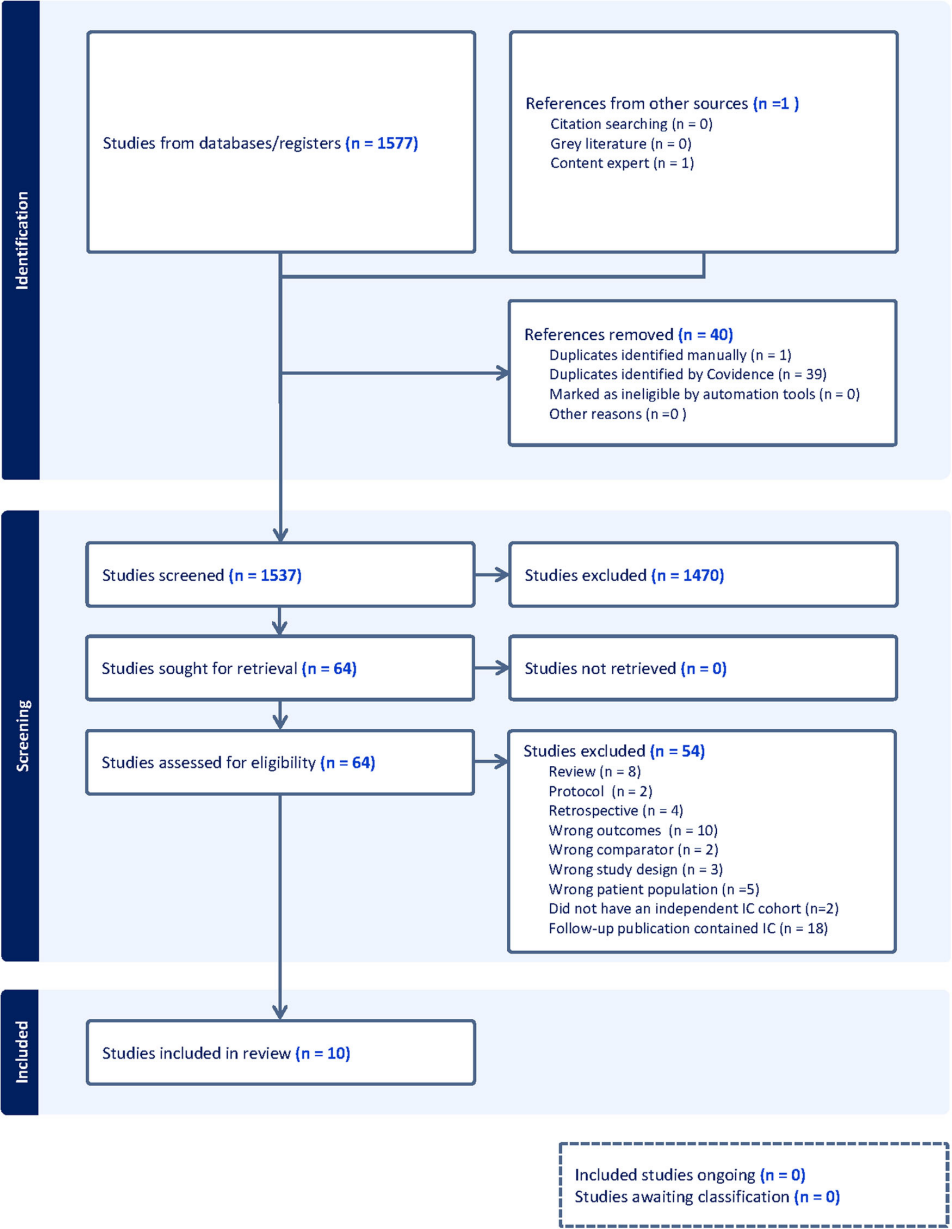
PRISMA flow diagram of included and excluded trials

Our search strategy (PRISMA diagram, Fig. 1) identified 1578 records of which 10 were eligible studies [18–27]. Characteristics of these studies are summarized in Table 1. All trials were prospective, three trials were RCTs and seven were non-randomised studies undertaken in population-based screening programmes (nine in Europe, one in Australia). All studies provided data for CDR, ICR and recall rates. All studies were ranked as low risk of bias on QUADAS-2 (Supplementary Table 2).

**Table 1:** Characteristics of eligible studies comparing DBT and DM

Trial name, (country)	RCT or non-randomised	Enrolment period	Screening interval, months	Age, (mean or median)	Inclusion criteria	Exclusion criteria	Screen reading DBT	Screen reading DM	DM comparison group
Córdoba Breast Tomosynthesis Screening Trial (CBTST) (Spain) [19]	Non randomised	Jan 2015–Dec 2016	24	58	Women aged 50–69 years.	Refusal to take part in the study.	Double reading, recalled if either screen positive: DBT + synthesized, DBT + synthesized + DM	Double reading, recalled if either screen positive	Women who studied in the same period were invited to the same screening programme. Two different DM devices.
BreastScreen Victoria Pilot (Australia) [18]	Non randomised	Aug 2017–Nov 2018	24	60	Targets women aged 50–74 years, but also allows women aged 40–49 years and older women. All women who for breast screening.	Women younger than 40 years; and who are unable to provide consent.	Double reading plus arbitration: DBT + synthesized	Double reading plus arbitration	Run in parallel from the same population, based on screen room availability or women who opted to have.
Malmö Breast Tomosynthesis Screening Trial (MBTST) (Sweden) [20]	Non randomised	Jan 2010–Feb 2015	24	56	For the DBT group: women aged 40–74 years living in Malmo, eligible for the Swedish BC screening programme. DM group: age-screen date matched control group: the same age at screening 1 year and the same date of screening 1 year per woman in the MBTST.	Pregnancy and women not speaking Swedish or English (DBT group) were not excluded from the contemporary DM group.	Double-read plus consensus meeting for scores above 3, DBT (MLO view)	Double-reading plus consensus meeting for scores above 3	The concurrent comparison group screened with DM-only from the same screening programme, age group, and the same timeframe.
Oslo Tomosynthesis Screening Trial (OTST) (Norway) [21]	Non randomised	DBT Nov. 22, 2010–Dec 2012 DM 2006–2009	24	59	Women aged 50–69, years selection based on the availability of radiographers and imaging systems.	Women who declined to undergo DBT, women who were not asked to participate because of limitations in the availability of radiographers/equipment, women scheduled who did not return, women with pacemakers, women unable to stand, and women with breast implants. All second examinations were excluded, including screening-detected malignancies (lymphomas and metastases), palpable BC and normal mammographic scores, and women with a screening-detected local recurrence.	DBT (One of the DBT double readings included FFDM + DBT and the other included synthesised + DBT), with consensus for scores 2–3	Double-reading with consensus for scores 2–3	Historical comparison group screened with DM-only from the same screening programme and age group [2006–2009]. The underlying risk of groups may differ as DM conducted in the three previous years to the DBT group.
Oslo-Vestfold-Vestre Viken study (OVVV) (Norway) [22]	Non randomised	Feb 2014–Jan 2016	24	59	Women aged 50–69 attended a population-based screening programme.	NA	Double reading with consensus; DBT + synthesized	Double reading with consensus	The concurrent comparison group screened with DM only from the same programme, age group, and timeframe DM screening (two locations) and DBT (a third location). The underlying risk of groups may differ as the screening modality received is determined by county residence.
Screening with Tomosynthesis OR standard Mammography trial (STORM) (Italy) [25]	Non randomised	Aug 2011–Jun 2012	24	58	Women aged 48 years or older who attended population-based screening.	NA	Sequential double-reading is recalled if either screen is positive; DBT + DM	Sequential double reading is recalled if either screen is positive	A concurrent group of women had attended the same screening services.
Trento pilot study (Italy) [26]	Non randomised	DBT: Oct 2014–Oct 2016 DM: Jan 2013–Oct 2014	24	58	Women aged 50 years who attended the Trento population-based screening.	NA	Double-reading plus arbitration; DBT + synthesised	Double-reading plus arbitration	A historical cohort of women who attended the programme. The underlying risk of groups may differ as the DM cohort was sourced from the year previous to DBT.
RETomo (Italy) [24]	RCT	Mar 2014–Aug 2017	12 Months for women aged 45–49 years and 24 months for women aged 50–69 years	55	Women aged 45–69 attending a screening in one of the three clinics with machines equipped with DBT for a new screening round. Only women who had already participated in the previous screening round were asked to participate.	Women aged 70–74 years were not included, previous BC, inclusion in or eligibility for hereditary BC surveillance programme, pregnancy, previous DBT examination, very large breasts, augmentation prostheses, or language barriers.	Double reading plus arbitration; DBT plus DM	Double reading plus arbitration	Randomised
Proteus Donna (Italy) [23]	Multicenter RCT	Dec 2014–Dec 2017	24	57	Women aged 46–68 years were invited to the regional BC screening programme. Attended BC screening at the study centres.	A personal history or symptoms of BC, oncological treatment for other malignancies, terminal or critical illness, inability to express informed consent and breast implants.	Double reading, recalled if either screen is positive; DBT plus DM	Double reading, recalled if either screen is positive	Randomised
To-Be (Norway) [17]	RCT	Jan 2017–Dec 2017	24	60	All women attending screening as a part of Breast Screen Norway. All women aged 50–69 years at average risk of BC biennial two view mammographic screening.	Those with breast implants.	Double reading with consensus, DBT + synthesised	Double reading with consensus	Randomised

### ICR

**Fig. 2:**
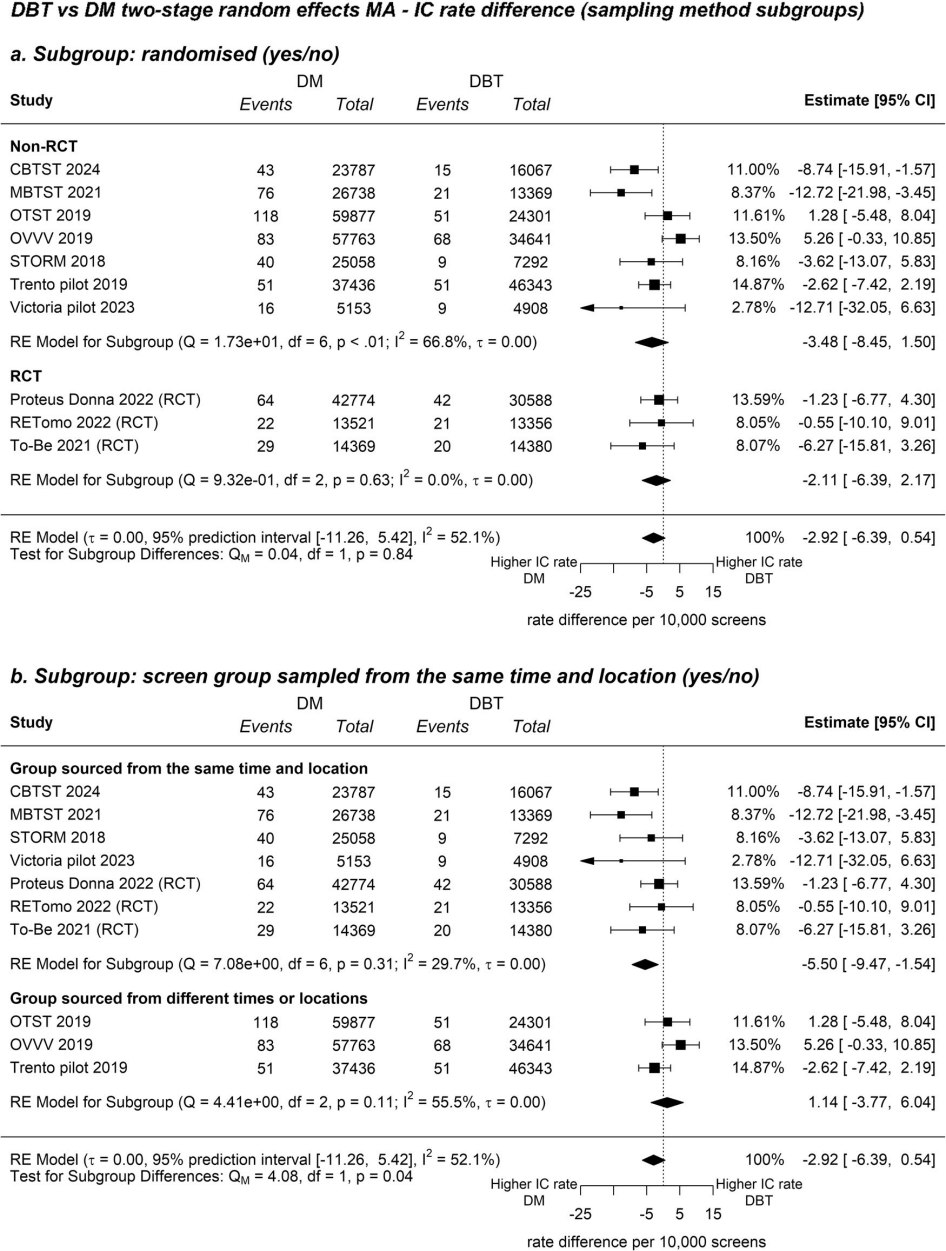
Sensitivity analyses of ICR difference per 10,000 screens. **a** ICR difference split by subgroup—RCT (yes/no). **b** ICR difference split by subgroup—group sourced from the same time and location (yes/no)

**Fig. 3:**
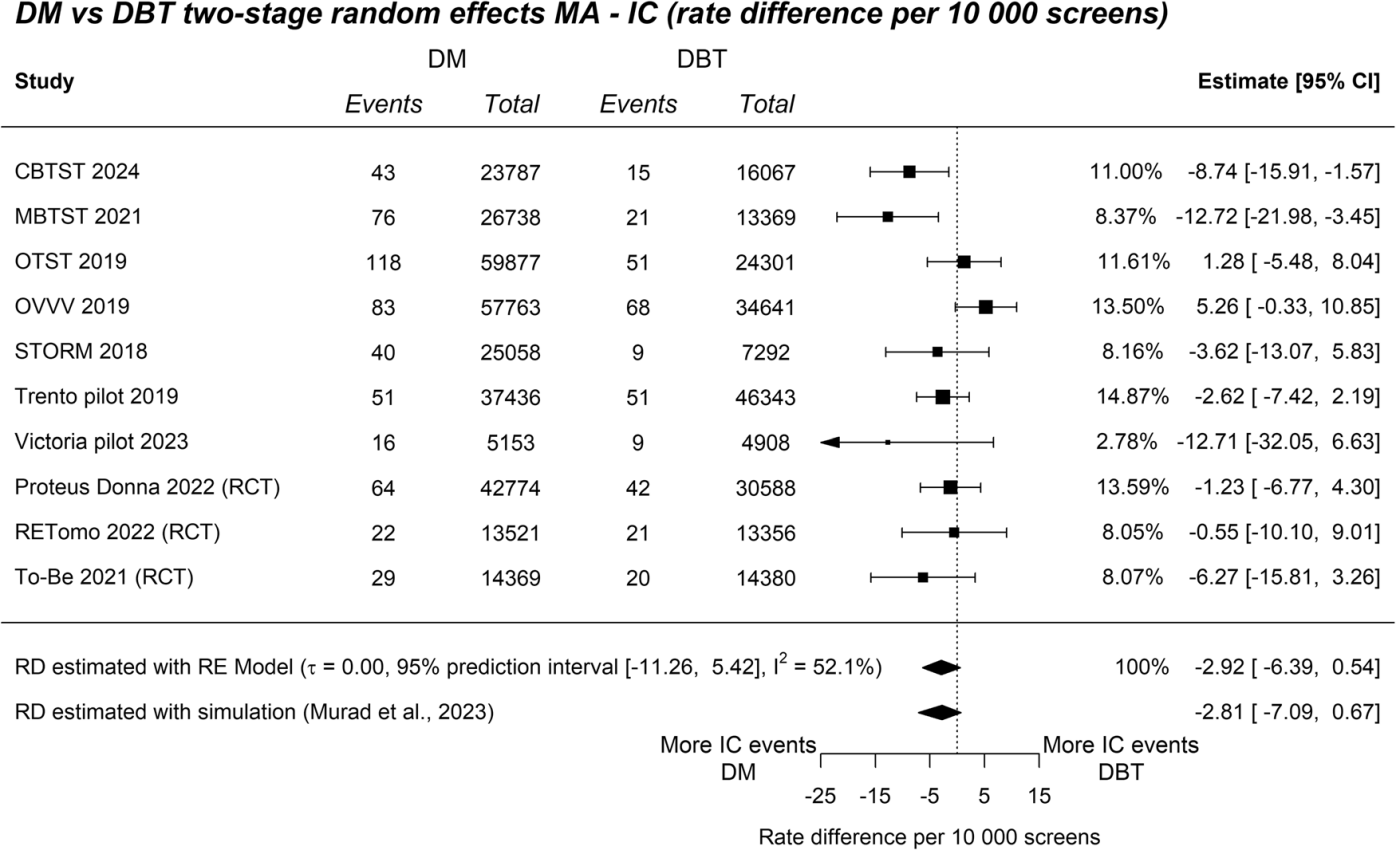
ICR difference per 10,000 screens. The top summary diamond represents the pooled RE model estimate. The bottom summary diamond represents the RD sensitivity estimate combining relative effects and the assumed baseline risk of DM using simulation (Murad et al [15]). CBTST, Córdoba breast tomosynthesis screening trial; Victoria Pilot, BreastScreen Victoria Pilot; MBTST, Malmö breast tomosynthesis screening trial; OTST, Oslo tomosynthesis screening trial; OVVV, Oslo–Vestfold–Vestre Viken study; STORM, screening with tomosynthesis or standard mammography trial

Across all studies, there were 307 interval cancers in 205,245 DBT-screened participants, and 542 interval cancers in 306,476 DM-screened participants. There was no evidence of a subgroup difference between RD in RCTs and non-randomised prospective studies for the outcome ICR (*Q*_M_ = 0.09, *p* = 0.76; Fig. 2a), thus we report the overall pooled estimate. The Pooled RR comparing DBT and DM for ICR was 0.84 (95% CI: 0.68–1.04), and there was high heterogeneity (95% PI: 0.51–1.39; *I*^2^ = 49.4%, Supplementary Fig. 4). The Pooled RD comparing DBT and DM for ICR per 10,000 screens was −2.92 (95% CI: −6.39 to 0.54), Fig. 3). Sensitivity analyses estimating RD using relative effects produced a similar point estimate (RD = −2.81 (95% CI: −7.09 to 0.67). Follow-up subgroup analyses compared studies that sourced groups from the same time and region, from those that did not, and indicated subgroups differed (*Q*_M_ = 5.45, *p* = 0.02; Supplementary Fig. 5). In the subgroup of trials that sampled groups from the same time and region, DBT produced a relative reduction in IC (RR: 0.72, 95% CI: 0.58–0.89, *I*^2^ = 6.6, 95% PI: 0.55–0.93, Supplementary Fig. 5) with a significant absolute reduction of −5.50 IC per 10,000 screens (95% CI: −9.47 to −1.54, *I*^2^ = 29.7, 95% PI: −12.39 to 1.38, Fig. 3B).

### CDR

**Fig. 4:**
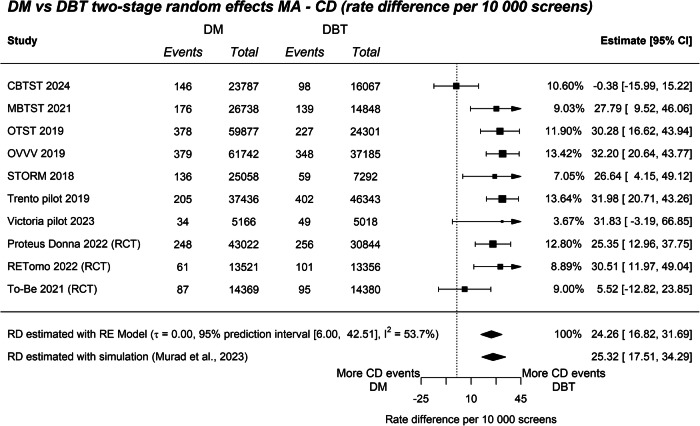
CDR difference per 10,000 screens. The top summary diamond represents the pooled RE model estimate. The bottom summary diamond represents the RD sensitivity estimate combining relative effects and the assumed baseline risk of DM using simulation (Murad et al [15]). CBTST, Córdoba breast tomosynthesis screening trial; Victoria Pilot, BreastScreen Victoria Pilot; MBTST, Malmö breast tomosynthesis screening trial; OTST, Oslo tomosynthesis screening trial; OVVV, Oslo–Vestfold–Vestre Viken study; STORM, screening with tomosynthesis or standard mammography trial

Across all studies 1774 cancers were detected in 209,634 DBT screens and 1850 cancers were detected in 310,716 DM screens. There was no evidence of a subgroup difference between the relative effects in RCTs and non-randomised prospective studies for CDR (*Q*_M_ = 0.09, *p* = 0.76; Supplementary Fig. 3), thus we report the overall pooled estimate. The pooled RR comparing the CDR of DBT and DM was 1.42 (95% CI: 1.30–1.55, Supplementary Fig. 3). The prediction interval (95% PI: 1.19–1.71, *I*^2^ = 35.8%) indicated, regardless of heterogeneity, that a new study would be expected to find DBT has a higher CDR than DM. The absolute CDR difference (RD) was 24.26 (95% CI: 16.82–31.69) per 10,000 screens (Fig. 4). The prediction interval (95% PI: 6.00–42.51) indicated that, regardless of heterogeneity, a new study would be expected to find DBT has a higher CDR than DM. Sensitivity analyses using relative effects produced comparable RD results (RD = 25.32; 95% CI: 17.51–34.29, Fig. 4). Subgroup analyses compared studies that sourced groups from the same time and region, from those that did not, and did not indicate subgroups significantly differed (*Q*_M_ = 3.52, *p* = 0.06; Supplementary Fig. 6).

### Recall rate

Collectively, 8416 of 209,636 DBT screens were recalled and 13,110 of 362,251 DM screens were recalled. There was no evidence of a subgroup difference between the relative effects in RCTs and non-randomised prospective studies for recall (*Q*_M_ = 0.16, *p* = 0.69; Supplementary Fig. 1), thus we pooled both study designs. There was no evidence of a difference in the recall rate between screening methods: RR = 1.04 (95% CI: 0.89–1.23, Supplementary Fig. 1). There was substantial heterogeneity (95% PI: 0.62–1.76, *I*^2^ = 96.9%). Absolute RD was 0.24 recalls per 100 (95% CI: −0.40 to 0.87, Supplementary Fig. 2) with substantial heterogeneity (95% PI: −1.82 to 2.29, *I*^2^ = 96.9%). Sensitivity analyses estimating RD using relative effects produced comparable results (RD = 0.18; 95% CI: −0.48 to 1.03, Supplementary Fig. 2). Follow-up subgroup analyses (studies that sourced groups from the same time/region vs those that did not) did not indicate subgroups significantly differed (*Q*_M_ = 2.66, *p* = 0.10; Supplementary Fig. 7).

## Discussion

In this systematic review and meta-analysis, we focus on prospective studies (including RCTs) reporting ICR at follow-up of participants screened with DBT vs DM to determine the effect on this key outcome in BC screening. Although earlier reviews have examined DBT screen detection [2, 4, 28], we address a critical evidence gap regarding ICR using high-level evidence (all prospective studies). Alongside DBT’s known effect of significantly increasing CDR [2, 4, 28], we report suggestive evidence that DBT may be associated with a reduction in ICR. Currently, many organised screening programmes conditionally recommend DBT instead of DM with very low certainty of evidence due to little or no effect on interval cancers. Our findings are timely and relevant to these screening practice recommendations [8].

The inclusion of both RCT and prospective non-randomised studies expanded the available sample size for evidence synthesis and enabled us to explore the potential for bias that may have been introduced in non-randomised studies. While we did not find DBT significantly reduced the ICR compared to DM, the estimate and CI provided an indication of a possible effect (pooled RD −2.92 per 10,000; 95% CI: −6.39 to 0.54)—this can be considered weak (suggestive) evidence of an effect. Although we did not find any evidence that non-randomised studies had bias for any outcome in our subgroup analyses, we did find evidence that studies that sourced cohorts from different periods or locations may have biased ICR estimates. Three studies sourced groups from different populations (this was done to allow comparison of ICR from independent groups): the OVVV study assigned screening modality based on the county of residence (DBT in Olso, DM in Vestfold and Vestre Viken), and the OTST and Trento pilot studies compared cohorts from different time periods (using historical cohorts). It has been well documented that BC incidence rates change across times and places in Europe [29, 30]. While incidence rates may be affected by many background factors, it is plausible that baseline ICR risk changes for groups sampled at different times and from different regions. A subgroup test revealed studies that sourced groups from the same period and region produced ICR outcomes that differed from studies that did not (*p* = 0.02). One explanation for the difference across subgroups is that sampling from different populations may lead to cohorts with different baseline cancer risks, which could bias within study estimates of ICR. Pooling the subgroup of studies that sampled groups from the same timeframe and region (*n* = 7), DBT produced a relative reduction in IC (RR: 0.72, 95% CI: 0.58–0.89, *I*^2^ = 6.6) with an absolute reduction of 5.50 IC per 10,000 screens (95% CI: −9.47 to −1.54, *I*^2^ = 29.7). Relative heterogeneity (as indicated by *I*^2^) reduced from 52% with all studies, to 29% when only including studies that sourced screening groups from the same population. This suggests studies that sourced cohorts from different timeframes and regions may have introduced substantial heterogeneity in estimates. It also indicates that DBT reduced the IC rate compared to DM when controlling for groups sampled from the same population. In interpreting these findings, it should be noted that the subgroup of studies that sampled from the same time and population (Fig. 2b), was an exploratory comparison and more evidence needs to accumulate to confirm this ICR finding.

Examining secondary outcomes confirms that DBT significantly increased CDR (pooled absolute RD 24.17/10,000 screens) vs DM, with consistent findings in subgroup analysis (by study design) and in sensitivity analysis. In contrast, there was no evidence of a difference in recall rate between DBT and DM in pooled analyses, and recall rates were heterogeneous across studies. These findings are consistent with previous reviews that have also found DBT has a higher CDR than DM, but mixed recall rates [2, 4, 27].

There is an increasing body of evidence on DBT’s greater CDR relative to DM particularly in biennial screening. This is in contrast to sparse information and inconclusive findings on the follow-up outcomes of DBT screened populations, specifically ICRs [11]. Such evidence is central to informing BC screening policy decisions. Interval BCs are diagnosed after a negative screen and have been found to share the prognostic features of clinically diagnosed cancers [9]. For these reasons, they are monitored as an indication of screening sensitivity and potential benefit [9, 31]. We have reported ICR as a surrogate measure for screening effect. In our main analysis, we return weak but inconclusive evidence that DBT reduces ICR relative to DM screening. However, in a post-hoc sensitivity analysis including only comparable populations, we report that DBT has a beneficial effect at follow-up by reducing ICR, meaning that DBT detected some of the cancers that would have clinically progressed within two years of screening, possibly contributing a mortality benefit. This suggests the increased CDR of DBT does not completely reflect over-detection [10]. While more evidence is needed to confirm any relative difference in ICR between screening methods, this review provides initial support for DBT in programmatic screening.

The review draws attention to possible bias introduced by sampling groups from different populations. While we cannot disentangle the true influence of all sources of bias, the difference in ICR results between trials that sourced groups from the same time and location and those that did not, presents suggestive evidence that such factors may influence ICR. Policymakers and practitioners may need to be mindful of such design features when using synthesised screening outcomes in decisions.

A limitation of our subgroup analyses (Fig. 2) is that subgroups were informed by the authors’ judgments about the most important potential sources of bias (whether randomisation was used; location or time differences across groups being compared), however, this approach is not exhaustive, and it is possible there are other sources of bias from known or unknown factors. Examples of possible sources of bias in specific trials are differences in reading strategies between arms in the Córdoba breast tomosynthesis screening trial (standard double-reading for DM and quadruple reading for DBT), and the 5% ‘opt-out’ from DBT in the BreastScreen Victoria pilot trial which meant that around 10% of women in the DM group chose not to receive DBT.

Previous studies have found that DBT may be more effective for dense breasts [32]. However, we did not attempt to analyse whether outcomes interact with breast density because only four of the eligible studies reported such data [18–20, 25]. Future reviews should consider using IPD meta-analysis or recovering additional aggregate information to enable the evaluation of whether breast density is an effect modifier of screening benefit. Another limitation of our review is the inability to directly evaluate overdiagnosis. Our findings indicate an additional 24 cancers are detected per 10,000 people using DBT, and that DBT may also produce approximately 5–3 fewer interval cancers per 10,000 screens during the biennial interval; if this is the case, a subset of the additional cancers detected by DBT did not progress to interval cancers within two years (so there may be some lead time effect) [10]. Longer-term follow-up data is needed to estimate overdiagnosis for DBT vs DM screening.

In conclusion, we present the initial but weak evidence that DBT is detecting additional clinically meaningful cancers. While this is a promising finding, future research is needed to quantify the longer-term benefits (beyond 2 years) and potential overdiagnosis.

## Supplementary information

Electronic Supplementary Material

## Abbreviations

BC: Breast cancer
CDR: Cancer detection rate
DBT: Digital breast tomosynthesis
DM: Digital mammography
ICR: Interval cancer rate
IPD: Individual participant data
RCT: Randomised controlled trials
RD: Rate differences
RR: Rate ratios

## References

[1] Li T, Marinovich ML, Houssami N (2018) Digital breast tomosynthesis (3D mammography) for breast cancer screening and for assessment of screen-recalled findings: review of the evidence. Expert Rev Anticancer Ther 18:785–791 29847744 10.1080/14737140.2018.1483243

[2] Libesman S, Zackrisson S, Hofvind S et al (2022) An individual participant data meta-analysis of breast cancer detection and recall rates for digital breast tomosynthesis versus digital mammography population screening. Clin Breast Cancer 22:e647–e654 35246389 10.1016/j.clbc.2022.02.005

[3] Ciatto S, Houssami N, Bernardi D et al (2013) Integration of 3D digital mammography with tomosynthesis for population breast-cancer screening (STORM): a prospective comparison study. Lancet Oncol 14:583–589 23623721 10.1016/S1470-2045(13)70134-7

[4] Marinovich ML, Hunter KE, Macaskill P, Houssami N (2018) Breast cancer screening using tomosynthesis or mammography: a meta-analysis of cancer detection and recall. J Natl Cancer Inst 110:942–949 30107542 10.1093/jnci/djy121

[5] Lång K, Andersson I, Rosso A, Tingberg A, Timberg P, Zackrisson S (2016) Performance of one-view breast tomosynthesis as a stand-alone breast cancer screening modality: results from the Malmö breast tomosynthesis screening trial, a population-based study. Eur Radiol 26:184–190 25929946 10.1007/s00330-015-3803-3 PMC4666282

[6] Houssami N (2022) Should tomosynthesis replace mammography for breast cancer screening? Lancet Oncol 23:554–555 35427472 10.1016/S1470-2045(22)00215-7

[7] Marmot MG, Altman D, Cameron D, Dewar J, Thompson S, Wilcox M (2013) The benefits and harms of breast cancer screening: an independent review. Br J Cancer 108:2205–2240 23744281 10.1038/bjc.2013.177 PMC3693450

[8] European Commission Initiative on Breast Cancer (ECIBC) (2023) European guidelines on breast cancer screening and diagnosis. 5. Use of Tomosynthesis. Available via https://cancer-screening-and-care.jrc.ec.europa.eu/en/ecibc/european-breast-cancer-guidelines?topic=65&usertype=60&updatef2=0

[9] Houssami N, Hunter K (2017) The epidemiology, radiology and biological characteristics of interval breast cancers in population mammography screening. NPJ Breast Cancer 3:12 28649652 10.1038/s41523-017-0014-x PMC5460204

[10] Farber R, Houssami N, Barnes I, McGeechan K, Barratt A, Bell KJ (2022) Considerations for evaluating the introduction of new cancer screening technology: use of interval cancers to assess potential benefits and harms. Int J Environ Res Public Health 19:14647 36429373 10.3390/ijerph192214647 PMC9691207

[11] Houssami N, Hofvind S, Soerensen AL et al (2021) Interval breast cancer rates for digital breast tomosynthesis versus digital mammography population screening: an individual participant data meta-analysis. EClinicalMedicine 34:100804 33997729 10.1016/j.eclinm.2021.100804 PMC8102709

[12] Akobeng AK (2005) Understanding randomised controlled trials. Arch Dis Child 90:840–844 16040885 10.1136/adc.2004.058222 PMC1720509

[13] Gardner MJ, Altman DG (1986) Confidence intervals rather than *P* values: estimation rather than hypothesis testing. Br Med J (Clin Res Ed) 292:746–750 3082422 10.1136/bmj.292.6522.746 PMC1339793

[14] Schünemann HJ, Higgins JP, Vist GE et al (2019) Completing ‘Summary of findings’ tables and grading the certainty of the evidence. In: Cochrane Handbook for systematic reviews of interventions. Wiley, Chichester, pp 375–402

[15] Murad MH, Wang Z, Zhu Y, Saadi S, Chu H, Lin L (2023) Methods for deriving risk difference (absolute risk reduction) from a meta-analysis. BMJ 381:e073141 37146999 10.1136/bmj-2022-073141

[16] Viechtbauer W (2010) Conducting meta-analyses in R with the metafor package. J Stat Softw 36:1–48

[17] Team RC (2024) R: a language and environment for statistical computing. R Foundation for Statistical Computing, Vienna. https://www.R-projectorg/

[18] Hofvind S, Moshina N, Holen ÅS et al (2021) Interval and subsequent round breast cancer in a randomized controlled trial comparing digital breast tomosynthesis and digital mammography screening. Radiology 300:66–76 33973840 10.1148/radiol.2021203936

[19] Houssami N, Lockie D, Giles M, Noguchi N, Marr G, Marinovich ML (2023) Two-year follow-up of participants in the BreastScreen Victoria pilot trial of tomosynthesis versus mammography: breast density-stratified screening outcomes. Br J Radiol 96:20230081 37191331 10.1259/bjr.20230081 PMC10392654

[20] Pulido-Carmona C, Romero-Martín S, Raya-Povedano JL et al (2024) Interval cancer in the Córdoba breast tomosynthesis screening trial (CBTST): comparison of digital breast tomosynthesis plus digital mammography to digital mammography alone. Eur Radiol 34:5427–5438 10.1007/s00330-023-10546-x PMC11255077 38177619

[21] Johnson K, Lång K, Ikeda DM, Åkesson A, Andersson I, Zackrisson S (2021) Interval breast cancer rates and tumor characteristics in the prospective population-based Malmö breast tomosynthesis screening trial. Radiology 299:559–567 33825509 10.1148/radiol.2021204106

[22] Skaane P, Sebuødegård S, Bandos AI et al (2018) Performance of breast cancer screening using digital breast tomosynthesis: results from the prospective population-based Oslo tomosynthesis screening trial. Breast Cancer Res Treat 169:489–496 29429017 10.1007/s10549-018-4705-2

[23] Hovda T, Holen ÅS, Lång K et al (2020) Interval and consecutive round breast cancer after digital breast tomosynthesis and synthetic 2D mammography versus standard 2D digital mammography in BreastScreen Norway. Radiology 294:256–264 31821118 10.1148/radiol.2019191337

[24] Armaroli P, Frigerio A, Correale L et al (2022) A randomised controlled trial of digital breast tomosynthesis vs digital mammography as primary screening tests: screening results over subsequent episodes of the Proteus Donna study. Int J Cancer 151:1778–1790 35689673 10.1002/ijc.34161

[25] Pattacini P, Nitrosi A, Giorgi Rossi P et al (2022) A randomized trial comparing breast cancer incidence and interval cancers after tomosynthesis plus mammography versus mammography alone. Radiology 303:256–266 35103537 10.1148/radiol.211132

[26] Houssami N, Bernardi D, Caumo F et al (2018) Interval breast cancers in the ‘screening with tomosynthesis or standard mammography’ (STORM) population-based trial. Breast 38:150–153 29328943 10.1016/j.breast.2018.01.002

[27] Bernardi D, Gentilini MA, De Nisi M et al (2020) Effect of implementing digital breast tomosynthesis (DBT) instead of mammography on population screening outcomes including interval cancer rates: results of the Trento DBT pilot evaluation. Breast 50:135–140 31607526 10.1016/j.breast.2019.09.012 PMC7375541

[28] Alabousi M, Zha N, Salameh J-P et al (2020) Digital breast tomosynthesis for breast cancer detection: a diagnostic test accuracy systematic review and meta-analysis. Eur Radiol 30:2058–2071 31900699 10.1007/s00330-019-06549-2

[29] van Luijt PA, Heijnsdijk EA, van Ravesteyn NT, Hofvind S, de Koning HJ (2017) Breast cancer incidence trends in Norway and estimates of overdiagnosis. J Med Screen 24:83–91 27754936 10.1177/0969141316668379

[30] Dafni U, Tsourti Z, Alatsathianos I (2019) Breast cancer statistics in the European Union: incidence and survival across European countries. Breast Care (Basel) 14:344–353 31933579 10.1159/000503219 PMC6940474

[31] Nederend J, Duijm LE, Louwman MW et al (2014) Impact of the transition from screen-film to digital screening mammography on interval cancer characteristics and treatment—a population-based study from the Netherlands. Eur J Cancer 50:31–39 24275518 10.1016/j.ejca.2013.09.018

[32] Li T, Houssami N, Noguchi N, Zeng A, Marinovich ML (2022) Differential detection by breast density for digital breast tomosynthesis versus digital mammography population screening: a systematic review and meta-analysis. Br J Cancer 127:116–125 35352019 10.1038/s41416-022-01790-x PMC9276736

