# Supplementary material for: Interval breast cancer rates for tomosynthesis vs mammography population screening: a systematic review and meta-analysis of prospective studies
Source: Eur Radiol. 2024 Oct 3;35(3):1478–89. doi: 10.1007/s00330-024-11085-9 (PMC11836155; doi:10.1007/s00330-024-11085-9)
Supplement: Supplementary file 1 — Electronic Supplementary Material [file 330_2024_11085_MOESM1_ESM.pdf]

Interval breast cancer rates for tomosynthesis versus mammography population screening: A systematic review and meta-analysis of prospective studies
ELECTRONIC SUPPLEMENTARY MATERIAL

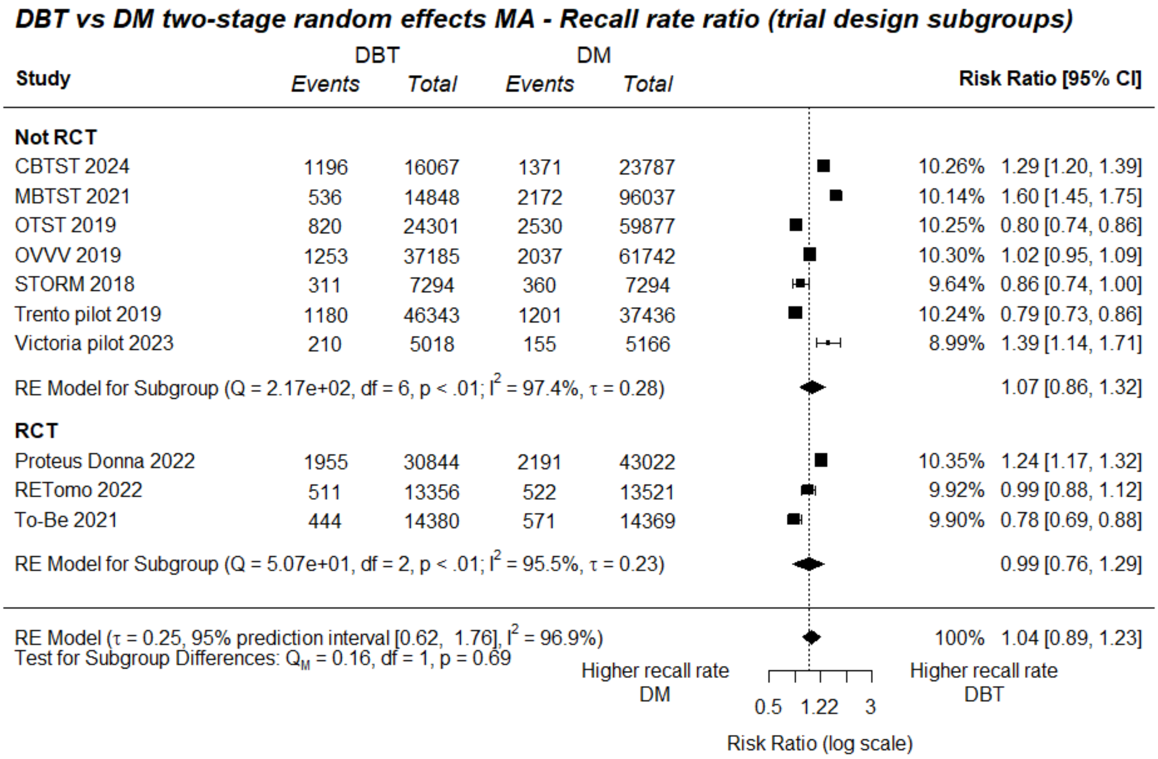

Supplementary Figure 1. Recall rate ratio split by subgroup RCT (yes/no)

DM vs DBT two-stage random effects MA - recall (rate difference per 100 screens)

Study	DM *Events*	DM *Total*	DBT *Events*	DBT *Total*	Weight	Estimate [95% CI]
CBTST 2024	1371	23787	1196	16067	9.86%	1.68 [1.18, 2.18]
MBTST 2021	2172	96037	536	14848	10.25%	1.35 [1.03, 1.66]
OTST 2019	2530	59877	820	24301	10.31%	-0.85 [-1.13, -0.57]
OVVV 2019	2037	61742	1253	37185	10.37%	0.07 [-0.16, 0.30]
STORM 2018 paired-data	360	7294	311	7294	9.38%	-0.67 [-1.35, 0.01]
Trento pilot 2019	1201	37436	1180	46343	10.37%	-0.66 [-0.89, -0.43]
Victoria pilot 2023	155	5166	210	5018	9.25%	1.18 [0.46, 1.91]
Proteus Donna 2022 (RCT)	2191	43022	1955	30844	10.20%	1.25 [0.90, 1.59]
RETomo 2022 (RCT)	522	13521	511	13356	9.96%	-0.03 [-0.49, 0.42]
To-Be 2021 (RCT)	571	14369	444	14380	10.04%	-0.89 [-1.31, -0.46]
RD estimated with RE Model (τ = 0.01, 95% prediction interval [-1.82, 2.29], I^2 = 96.9%)					100%	0.24 [-0.40, 0.87]
RD estimated with simulation (Murad et al., 2023)						0.18 [-0.48, 1.03]

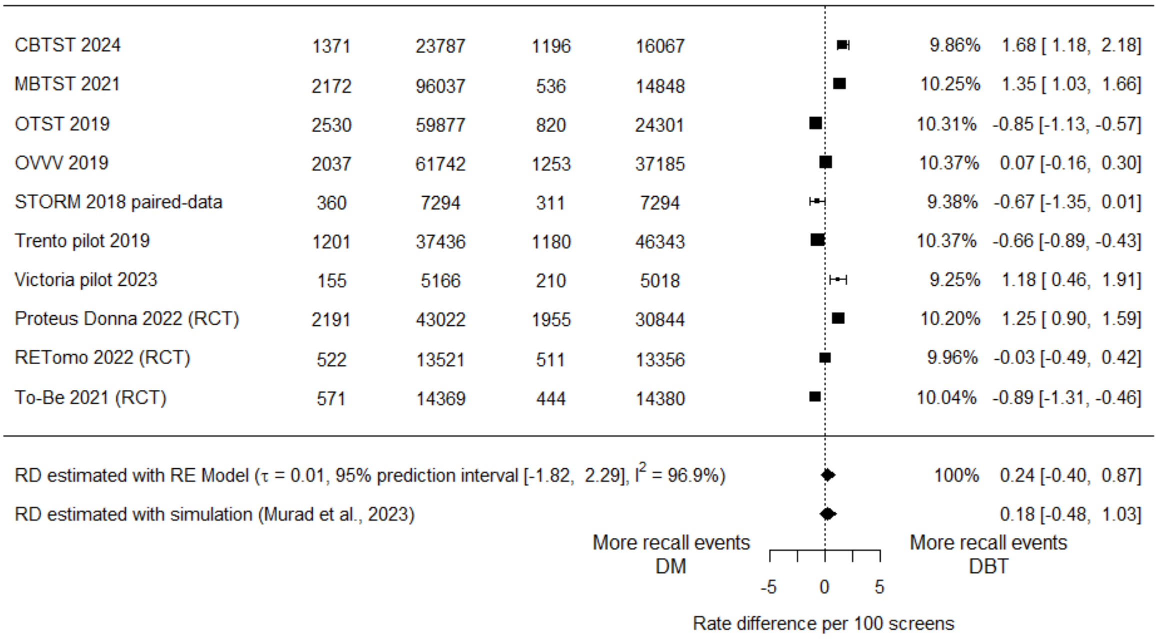

Supplementary Figure 2. Recall risk difference per 100 screens. The top summary diamond represents the pooled RE model estimate. The bottom summary diamond represents the RD sensitivity estimate combining relative effects and the assumed baseline risk of DM using simulation (Murad et al., 2023).

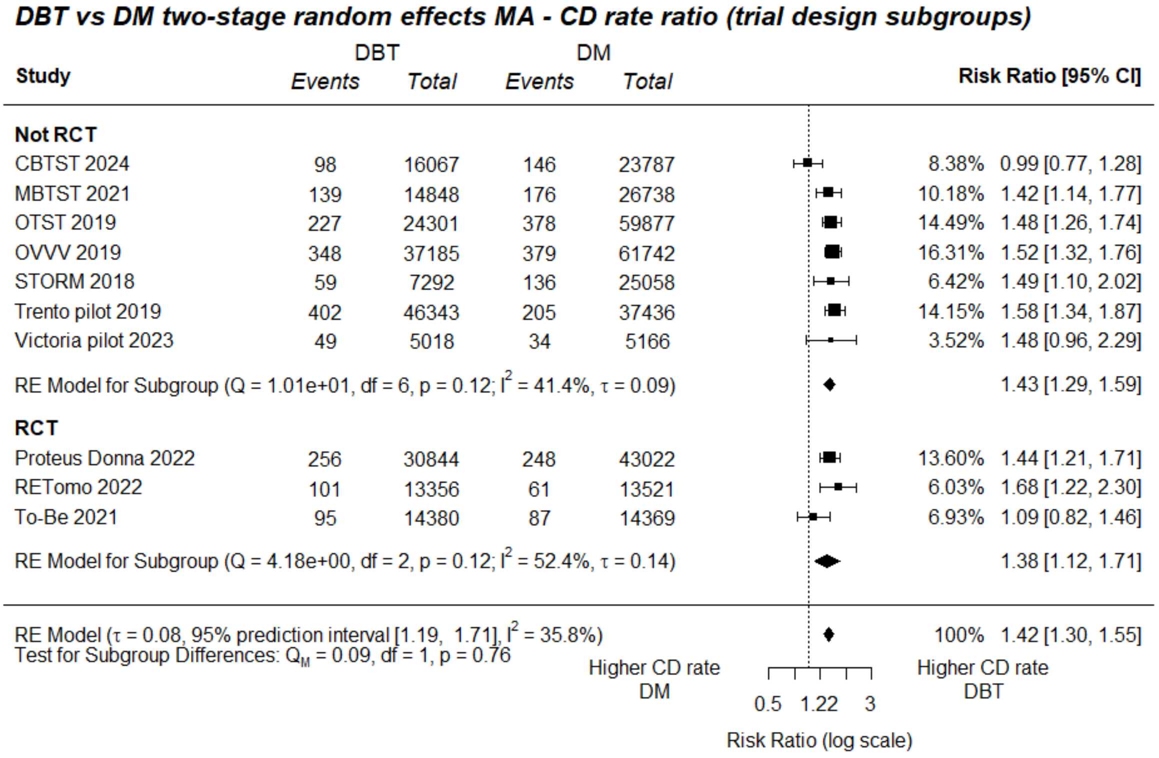

Supplementary Figure 3. Cancer detected rate ratio split by subgroup RCT (yes/no)

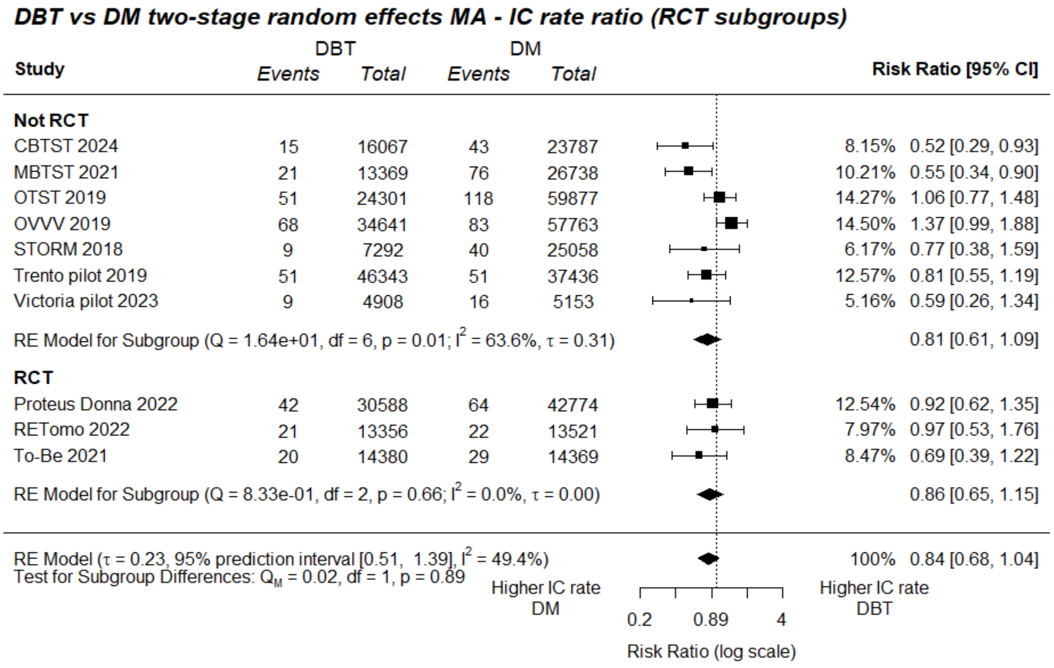

Supplementary Figure 4. Interval Cancer rate ratio split by subgroup RCT (yes/no).

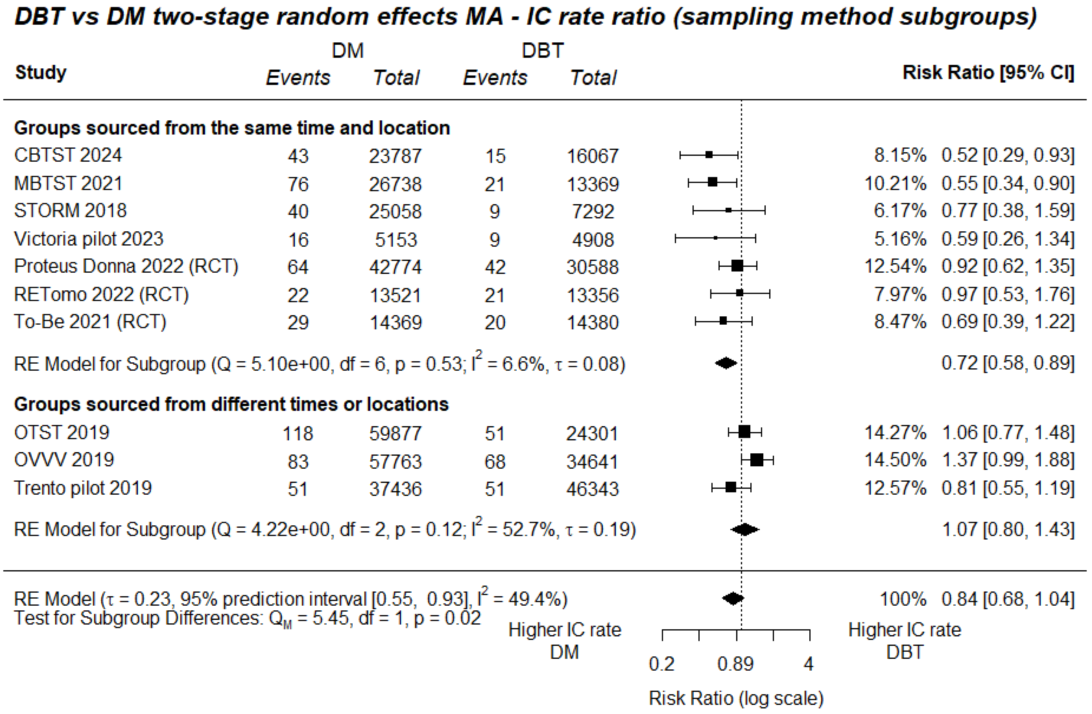

Supplementary Figure 5. Interval cancer rate ratio split by the cohort sampling method (were screening groups sourced from the same time period/location-(yes/no)

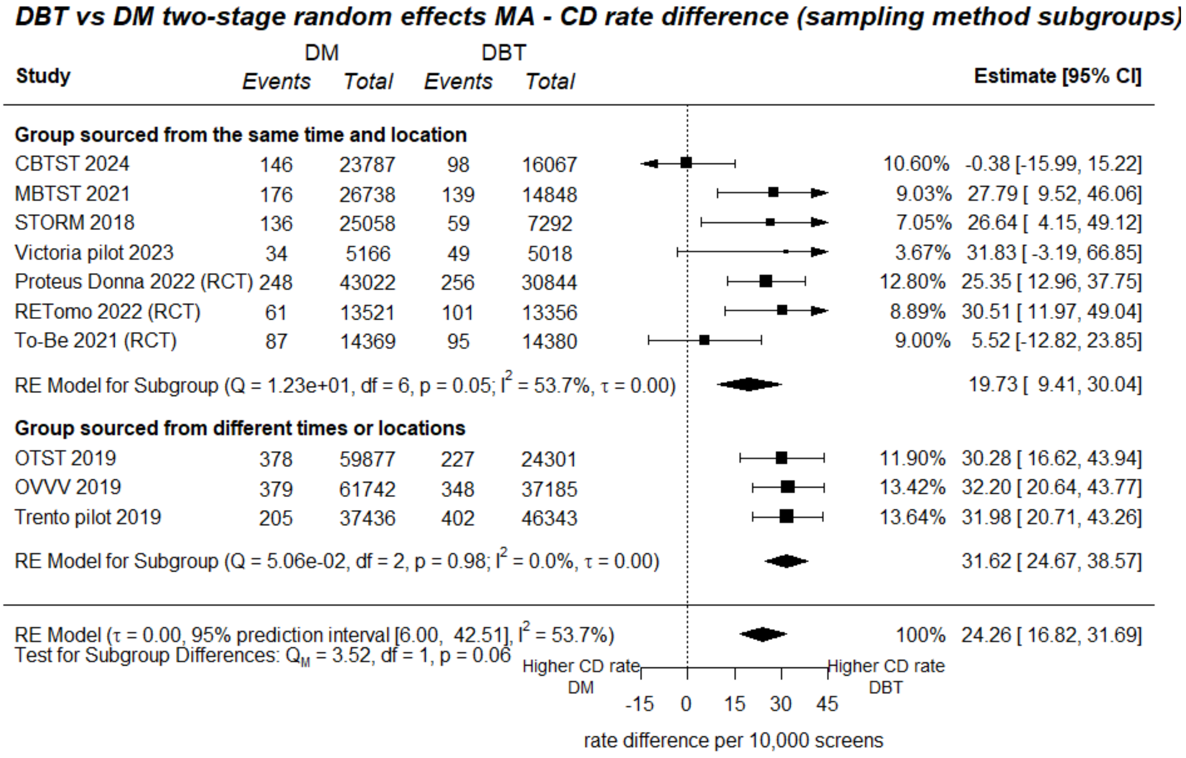

Supplementary Figure 6. Cancer detected rate difference per 10,000 split by the cohort sampling method (were screening groups sourced from the same time period/location-(yes/no).

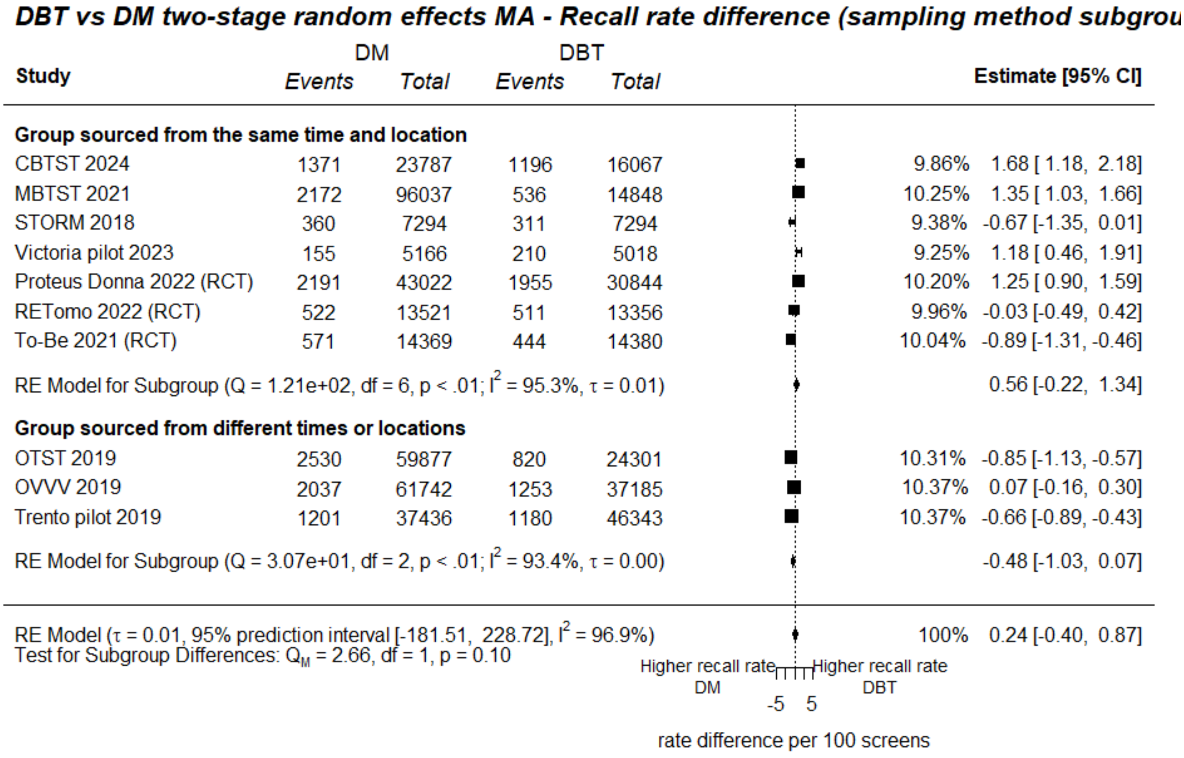

Supplementary Figure 7. Recall rate difference per 100 split by the cohort sampling method (were screening groups sourced from the same time period/location-(yes/no).

Supplementary Table 1. PRISMA 2020 Checklist

Section and Topic	Item #	Checklist item	Location where item is reported
**TITLE**			
Title	1	Identify the report as a systematic review.	1
**ABSTRACT**			
Abstract	2	See the PRISMA 2020 for Abstracts checklist.	1
**INTRODUCTION**			
Rationale	3	Describe the rationale for the review in the context of existing knowledge.	2
Objectives	4	Provide an explicit statement of the objective(s) or question(s) the review addresses.	2
**METHODS**			
Eligibility criteria	5	Specify the inclusion and exclusion criteria for the review and how studies were grouped for the syntheses.	2-3
Information sources	6	Specify all databases, registers, websites, organisations, reference lists and other sources searched or consulted to identify studies. Specify the date when each source was last searched or consulted.	3
Search strategy	7	Present the full search strategies for all databases, registers and websites, including any filters and limits used.	3
Selection process	8	Specify the methods used to decide whether a study met the inclusion criteria of the review, including how many reviewers screened each record and each report retrieved, whether they worked independently, and if applicable, details of automation tools used in the process.	3
Data collection process	9	Specify the methods used to collect data from reports, including how many reviewers collected data from each report, whether they worked independently, any processes for obtaining or confirming data from study investigators, and if applicable, details of automation tools used in the process.	3
Data items	10a	List and define all outcomes for which data were sought. Specify whether all results that were compatible with each outcome domain in each study were sought (e.g. for all measures, time points, analyses), and if not, the methods used to decide which results to collect.	3
	10b	List and define all other variables for which data were sought (e.g. participant and intervention characteristics, funding sources). Describe any assumptions made about any missing or unclear information.	3
Study risk of bias assessment	11	Specify the methods used to assess risk of bias in the included studies, including details of the tool(s) used, how many reviewers assessed each study and whether they worked independently, and if applicable, details of automation tools used in the process.	4
Effect measures	12	Specify for each outcome the effect measure(s) (e.g. risk ratio, mean difference) used in the synthesis or presentation of results.	4
Synthesis methods	13a	Describe the processes used to decide which studies were eligible for each synthesis (e.g. tabulating the study intervention characteristics and comparing against the planned groups for each synthesis (item #5)).	4, Fig 1.
	13b	Describe any methods required to prepare the data for presentation or synthesis, such as handling of missing summary statistics, or data conversions.	NA
	13c	Describe any methods used to tabulate or visually display results of individual studies and syntheses.	4
	13d	Describe any methods used to synthesize results and provide a rationale for the choice(s). If meta-analysis was performed, describe the	4

Section and Topic	Item #	Checklist item	Location where item is reported
		model(s), method(s) to identify the presence and extent of statistical heterogeneity, and software package(s) used.	
	13e	Describe any methods used to explore possible causes of heterogeneity among study results (e.g. subgroup analysis, meta-regression).	4
	13f	Describe any sensitivity analyses conducted to assess robustness of the synthesized results.	4
Reporting bias assessment	14	Describe any methods used to assess risk of bias due to missing results in a synthesis (arising from reporting biases).	4
Certainty assessment	15	Describe any methods used to assess certainty (or confidence) in the body of evidence for an outcome.	5
**RESULTS**			
Study selection	16a	Describe the results of the search and selection process, from the number of records identified in the search to the number of studies included in the review, ideally using a flow diagram.	Fig 1.
	16b	Cite studies that might appear to meet the inclusion criteria, but which were excluded, and explain why they were excluded.	Fig 1.
Study characteristics	17	Cite each included study and present its characteristics.	4
Risk of bias in studies	18	Present assessments of risk of bias for each included study.	4
Results of individual studies	19	For all outcomes, present, for each study: (a) summary statistics for each group (where appropriate) and (b) an effect estimate and its precision (e.g. confidence/credible interval), ideally using structured tables or plots.	4-5
Results of syntheses	20a	For each synthesis, briefly summarise the characteristics and risk of bias among contributing studies.	4
	20b	Present results of all statistical syntheses conducted. If meta-analysis was done, present for each the summary estimate and its precision (e.g. confidence/credible interval) and measures of statistical heterogeneity. If comparing groups, describe the direction of the effect.	4-5
	20c	Present results of all investigations of possible causes of heterogeneity among study results.	4-5
	20d	Present results of all sensitivity analyses conducted to assess the robustness of the synthesized results.	4-5
Reporting biases	21	Present assessments of risk of bias due to missing results (arising from reporting biases) for each synthesis assessed.	4
Certainty of evidence	22	Present assessments of certainty (or confidence) in the body of evidence for each outcome assessed.	NA
**DISCUSSION**			
Discussion	23a	Provide a general interpretation of the results in the context of other evidence.	5-6
	23b	Discuss any limitations of the evidence included in the review.	6
	23c	Discuss any limitations of the review processes used.	6
	23d	Discuss implications of the results for practice, policy, and future research.	6
**OTHER INFORMATION**			
Registration and protocol	24a	Provide registration information for the review, including register name and registration number, or state that the review was not registered.	2
	24b	Indicate where the review protocol can be accessed, or state that a protocol was not prepared.	2
	24c	Describe and explain any amendments to information provided at registration or in the protocol.	2
Support	25	Describe sources of financial or non-financial support for the review, and the role of the funders or sponsors in the review.	7
Competing	26	Declare any competing interests of review authors.	7

Section and Topic	Item #	Checklist item	Location where item is reported
interests			
Availability of data, code and other materials	27	Report which of the following are publicly available and where they can be found: template data collection forms; data extracted from included studies; data used for all analyses; analytic code; any other materials used in the review.	NA

From: Page MJ, McKenzie JE, Bossuyt PM, Boutron I, Hoffmann TC, Mulrow CD, et al. The PRISMA 2020 statement: an updated guideline for reporting systematic reviews. BMJ 2021;372:n71. doi: 10.1136/bmj.n71

For more information, visit: http://www.prisma-statement.org/

Supplementary Table 2. Quality appraisal using the revised Quality Assessment of Diagnostic Accuracy Studies (QUADAS-2) (Whiting et al 2011) checklist, as adapted by Marinovich et al. 2018 for application to studies in the breast cancer screening setting.

**Study**	**Selection**				**Index test**		**Reference Standard**		**Patient flow**			
	Risk of bias 1 = Low, 2 = High, 3 = Unclear				Risk of bias 1 = Low, 2 = High 3 = Unclear		Risk of bias 1 = Low, 2 = High, 3 = Unclear		Risk of bias 1 = Low, 2 = High, 3 = Unclear			
	Was a consecutive or random sample of patients enrolled?	Was a case-control design avoided?	Did the study avoid inappropriate exclusions?	Are there concerns that included patients and setting do not match the review question?	If a threshold or rule for recall was used, was it pre-specified?	Are there concerns that the index test, its conduct, or interpretation differ from the review question?	Is the reference standard likely to correctly classify the target condition?	Are there concerns that the target condition as defined by the reference standard does not match the question?	Did all patients receive a reference standard?	Did all RECALLED patients receive a reference standard?	Did all RECALLED patients receive the same reference standard?	Were all patients included in the analysis?
STORM	1	1	1	1	1	1	1	1	1	1	1	1
MBTST	1	1	1	1	1	1	1	1	1	1	1	1
STORM-2	1	1	1	1	1	1	1	1	1	1	1	1
OVVV	1	1	1	1	1	1	1	1	1	1	1	1
OTST	1	1	1	1	1	1	1	1	1	1	1	1
Trento pilot	1	1	1	1	1	1	1	1	1	1	1	1
Victoria pilot	1	1	1	1	1	1	1	1	1	1	1	1

Proteus Donna	1	1	1	1	1	1	1	1	1	1	1	1
RETomo	1	1	1	1	1	1	1	1	1	1	1	1
To-Be	1	1	1	1	1	1	1	1	1	1	1	1
CBTST	1	1	1	1	1	1	1	1	1	1	1	1

Whiting PF, Rutjes AW, Westwood ME, Mallett S, Deeks JJ, Reitsma JB, Leeflang MM, Sterne JA, Bossuyt PM, QUADAS-2 Group*. QUADAS-2: a revised tool for the quality assessment of diagnostic accuracy studies. Annals of internal medicine. 2011 Oct 18;155(8):529-36.

Marinovich ML, Hunter KE, Macaskill P, Houssami N. Breast cancer screening using tomosynthesis or mammography: a meta-analysis of cancer detection and recall. J Natl Cancer Inst 2018;110:942–9.

Supplementary Table 3: Search Strategy

Ovid search terms for EMBASE, PREMEDLINE , DARE, HTA, NHSEED, ACP Journal Club, Cochrane
1. tomosynthesis.mp<br>2. dbt.mp<br>3. 3d mammogra*.mp<br>4. exp digital breast tomosynthesis/<br>5. or/1-4<br><br>6. breast.mp<br>7. exp breast cancer/<br>8. exp breast/<br>9. or/6-8<br><br>10. screening.mp<br>11. exp screening/<br>12. exp early cancer diagnosis/<br>13. or/10-12<br><br>14. 5 and 9 and 13<br><br>15. limit 14 to yr="2013 -Current"
